# Children and neonates anesthesia in magnetic resonance environment in Italy: an active call survey

**DOI:** 10.1186/s12871-022-01821-3

**Published:** 2022-09-02

**Authors:** Fabio Sbaraglia, Giorgia Spinazzola, Alessia Adduci, Nicola Continolo, Mariella De Riso, Giuliano Ferrone, Rossano Festa, Rossella Garra, Federica Tosi, Marco Rossi

**Affiliations:** Department of Anesthesia and Intensive Care, Fondazione Policlinico Universitario Agostino Gemelli IRCCS, Università Cattolica del Sacro Cuore, Largo F. Vito 1, 00168 Rome, Italy

**Keywords:** Magnetic Resonance, Anesthesia, Sedation, Neonates, Children, Monitoring

## Abstract

**Background:**

Pediatric anesthesia care in the Magnetic Resonance Imaging is a challenge for clinicians. The recent debate about the role of anesthetic agent on neural development, encouraged an evaluation of their actual activity in this environment. In this active call survey, the authors sought to delineate the Italian situation regarding national centers, staff involved, monitoring tools available and sedation techniques.

**Methods:**

A complete sample of all national centers performing almost a pediatric discharge in the 2014 was obtained from Health Ministry registers. All Institutions were contacted for a prospective phone investigation and a three-section survey was fill out with the Physician in charge. A descriptive and exploratory analyzes about the organization setting of the Centers were performed.

**Results:**

Among 876 Institution screened, only 106 (37%) met minimal criteria for inclusion. Children are managed by anesthesiologists in the 95% of cases, while neonates in the 54%. A dedicated nurse is present in 74% of centers. While a pulse oximetry is present in 100% of centers, the rate of prevalence of other monitoring is lower. A specific MRI-compatible ventilator is available in the 95% of Centers, but many tools are not equally homogenously distributed. Pharmacological approach is preferred in pediatric age (98%), but its use for newborns is reduced to 43%.

**Conclusions:**

We found significant heterogeneity in the daily clinical practice of sedation in MRI. Our results could be a starting point to evaluate the further evolution of approach to children and neonates in magnetic resonance setting.

**Trial registration:**

ClinicalTrials.gov identifier: NCT04775641.

**Supplementary Information:**

The online version contains supplementary material available at 10.1186/s12871-022-01821-3.

## Introduction

Diagnostic radiology procedures in childhood are increasingly required, above all for neurological assessment [1]. Magnetic Resonance Imaging (MRI) is an expanding imaging modality, and is currently the first choice for many instances in pediatric patients, both children and neonates [2, 3].

In order to provide a high-quality resolution, MRI in pediatric patients requires prolonged immobilization, most of procedures in that setting are managed under deep sedation [4].

A particular skillness is needed in this field, as well as a dedicated organization to ensure maximal efficiency, and at the same time safety for the patients. Among the goals of the anesthesia care, there is mainly the aim to minimize physical discomfort and psychological trauma [5]. The therapeutic window between sedation and anesthesia is very narrow for pediatric patients, and the burden of adverse events not trivial [6]. Moreover, the recent debate about neurotoxicity of anesthetics in developing age [7, 8], prompted the anesthesiological community to produce many papers and reviews about the role of the anesthetists during MRI for children and neonates [9, 10].

Despite a wide literature, evidence in this field is very poor, and there is not clear information about the preferred approaches, tools and techniques [11]. The absence of prospective data about pediatric and neonatal MRI casts many doubts on their gold standard. To finding the optimal balance between short term safety (periprocedural adverse events), long term damage (possible neurotoxicity of anesthetic agents) and best quality in imaging, could be particularly hard in such hostile environment. We need dedicated monitoring and tools, due to several technical restriction, but above all we should find the pharmacological approach able to minimize neurocognitive damages in the developing brain [12]. It is out of doubt, the needing of a pivotal study, to identify the state of art to ensure safe steps forward a good clinical practice [13]. For this reason, an active Italian Survey to take a stock of situation about clinical organization models and first choice techniques for the management of uncooperative children and neonates during MRI, was promoted. The survey was designed to capture the clinical practice in both neonatal and pediatric patients. It is meant to be a first step towards the identification of shared anesthesia protocols for the safe management of pediatric and neonatal patients scheduled for MRI procedures.

## Methods

In order to identify a complete sample of all the national Institutions that performed pediatric activity, we used data sourced from Italian Health Ministry Register referred to surgical and not surgical admission of pediatric patients in the 2014 [14], and endorsed by the Italian Society of Pediatric and Neonatal Anesthesia (SARNePI).

**Fig. 1:**
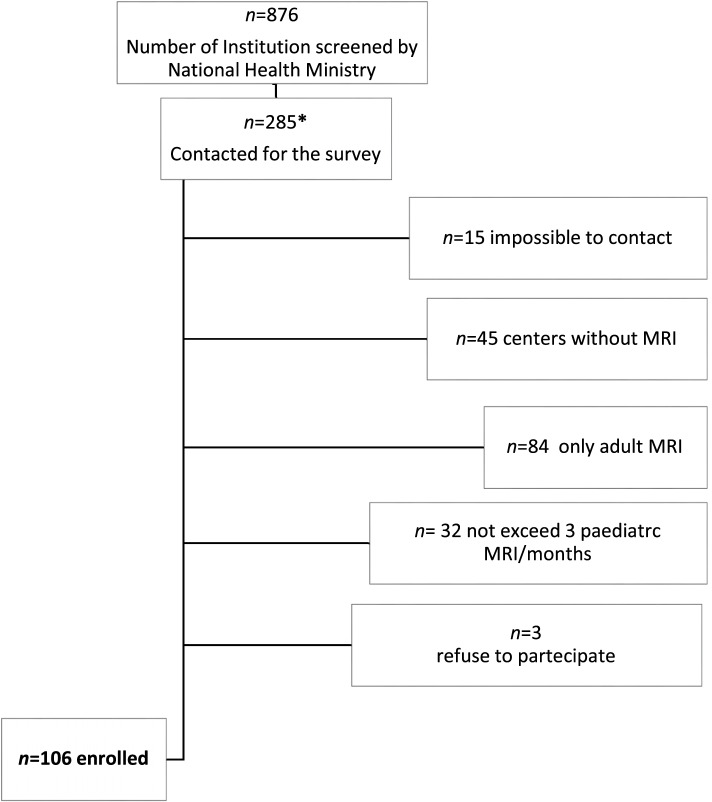
Flow Chart. * Centers performing at least a pediatric discharge in the 2014

We included all uncooperative children aged from the birth up to 14 years old and Neonatal Intensive Care Unit (NICU) neonates, scheduled for a specified pediatric anesthesia activity for MRI (Fig. 1). Once the recruitable centers were identified, a telephone investigation was launched. According to the study design, we established a cut-off of at least three procedures a month in order to exclude centers with just a sporadic activity.

In the following step, the physician usually in charge for pediatric anesthesia in radiological settings was contacted for every enrolled Center by a member of the investigational board. After obtaining verbal consent to participate, a three Section Survey was then exposed by phone, and the answers collected in apposite computerized sheets. There was no incentive to participate and referents were free to decline to participate to the survey or withdraw at any time.

The three Section model includes: Logistic organization, Pediatric management and NICU’s neonates management (Additional file 1). In this article, the description of logistics organization, anesthesia technique, drugs and airways device were reported.

### Statistical analysis

Data were analyzed using MEDcalc version 18.6. Before starting data analysis to address study aims, descriptive and exploratory analyzes were performed to identify any data anomalies, such as missing data or outliers (which may be related to data entry errors or invalid responses). Data from the survey were summarized using simple descriptive statistics including mean (standard deviation), n (%) and range. Proportions were compared via Chi square or Fisher’s exact test for equal proportions.

## Results

### Centers and procedures

Among 876 Institution only 106 (37%) met minimal criteria for MRI procedures on pediatric patients, and were enrolled in the survey, (Fig. 1).

Among the 106 Centers included in the Survey, 62% (*n* = 65) provided both children and NICU neonates MRI (Group A), while 38% (*n* = 41) performed anesthesia assistance only in children (Group B).

We have arbitrarily divided institutions enrolled in the survey into High Volume (HV) and Low Volume (LV) Centers, according to the number of procedures performed each week (≥ or < 10 procedures respectively for children; ≥ or < 3 procedures respectively for neonates).

**Fig. 2:**
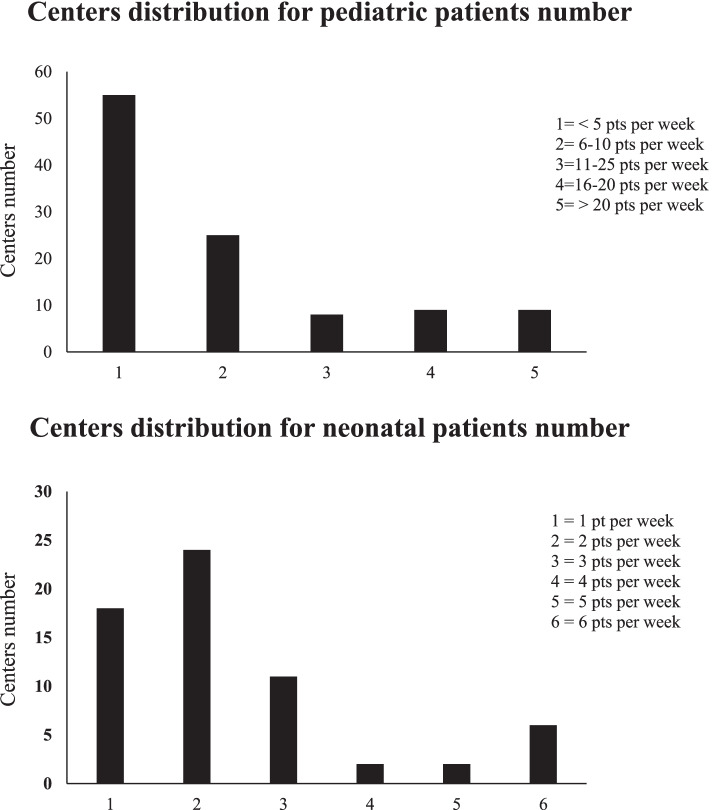
Centers Distribution

As showed in Fig. 2, 80 centers performed less than 10 MRI procedures per week; while 26 centers performed more than 10 MRI procedures per week. Referring to NICU neonates, 53 centers performed less than 3 MRI procedures per week, while 12 centers performed more than 3 procedures MRI per week. The Italian Health System provides admittance for pediatric MRI as inpatient with overnight (IN), day hospital with admission and discharge in the same day (DH), or outpatients with discharge directly by MRI suite (OUT).

Most of the centers (62%, *n* = 66) included the three ways of admittance. The remaining 21% (*n* = 23) showed an exclusive choice (respectively: IN 11, DH 10, OUT 2), and the 16% (*n* = 17) a preferring modality in > 90% of cases (respectively: IN 5, DH 11, OUT 1).

### Medical staff

Children are managed by anesthetists in 75 centers (71%), by a pediatrician in 27 centers (26%), by a cooperation between both physicians in 4 center (3%) (Table 1). Considering only the Group B (centers treating only children) the rate of anesthesiologists reaches 95% (*n* = 39/41).

**Table 1:** Physicians involved during sedation in MRI

	Centers with sedations in neonates and children (Group A ***n*** = 65)	Centers with sedations only in children (Group B ***n*** = 41)	***P***	All Centers (***n*** = 106)
**Anaesthesiologists**	54%	95%	< 0.01	71%
**Paediatricians**	40%	5%	0.01	26%
**Both anaesthesiologists and Paediatricians**	6%	0%	< 0.01	3%

Group A: Centers that provided anaesthesia assistance both children and NICU neonates for MRI, Group B: Centers that provided anaesthesia assistance only in children for MRI

For the NICU’s patients the preferred attendant is the anesthesiologist in 35 Institutions (54%), the neonatologist in 26 centers (40%), while a cooperation between both the specialists was found in 4 centers (6%).

A dedicated nurse is present in 79 centers (74%), as overall data, both during the procedure and in the Recovery Area after MRI. In Group A the percentage goes down until 72%, increasing to 78% in the Group B (*P* = 0.51).

### Monitoring

As showed in Table 2, Pulse oximetry is the only monitoring system available in all the settings, while Blood Pressure measurement tolls are less prevalent (65%). In the Recovery Area after MRI, the availability of monitoring systems is reduced of few points. No significant differences were detected between the Group A and B for monitoring availability during and after the MRI procedure.

**Table 2:** Monitoring tolls during sedation in MRI

		Centers with sedations in all patients (Group A ***n*** = 65)	Centers with sedations only in children (Group B ***n*** = 41)	P	All Centers (***n*** = 106)
**SpO**_**2**_	*inside*	100%	100%	0.47	100%
	*outside*	92%	98%	0.47	94%
**EKG**	*inside*	92%	98%	0.50	94%
	*outside*	88%	95%	0.29	91%
**EtCO**_**2**_	*inside*	82%	78%	0.85	83%
	*outside*	75%	73%	0.87	74%
**BP**	*inside*	66%	61%	0.86	65%
	*outside*	65%	63%	0.76	66%

*Abbreviations*: *SpO*_*2*_ oxygen peripheral saturation, *EKG* electrocardiogram, *EtCO*_*2*_ End-tidal carbon dioxide, *BP* blood pressure

### Tools

In Table 3 is reported the availability of anesthetic machines and instruments is reported. A MRI compatible ventilator is present in 97% of Centers of Group A, and in 93% in Group B, provided of a specific vaporizer for Sevoflurane in 86 and 83% of the Groups respectively. A gas scavenging system was implemented in 75% of MRI room in Group A and in 68% in Group B, while a suction device was available in 98% and in 90% respectively. Non-magnetic infusion pumps (or non- magnetic protecting box) were provided in 37% of the centers where anesthesia/sedation is performed for both children and neonates, and in 39% of Institutions of Group B. The queries of the Survey did not ask for the use of drugs administration systems outside the MRI.

**Table 3:** Anesthesiologic tools during sedation in MRI

	Centers with sedations in all patients (Group A ***n*** = 65)	Centers with sedations only in children (Group B ***n*** = 41)	P	All Centers (***n*** = 106)
**Mechanical ventilator**	97%	93%	0.33	95%
**Anaesthetic vaporizer**	86%	83%	0.15	82%
**Scavenger**	75%	68%	0.34	72%
**Infusion pumps**	37%	39%	0.88	38%
**Suction devices**	98%	90%	0.04	95%

### Children sedation techniques

When pediatric patients are treated (Table 4), non-pharmacological approach is very uncommon (2%), and in 38% of cases a premedication was administered. The drugs chosen for sedation are equally distributed between intravenous (44%) and volatile (40%) agents. The airways management is favorable forward less invasive method with the large use of external devices (78%) versus laryngeal mask (22%) or endotracheal intubation (0%).

**Table 4:** First choice Sedation technique in pediatric centers

Sedation in pediatric centers n. 106			
**Drug Sedation**	**Yes n. 104 (98%)**		**None n. 2 (2%)**
	Sevoflurane n. 40 (38%)	Propofol n. 34 (33%)	
	Thiopental n. 11 (11%)	Multidrugs^a^ n. 19 (18%)	
**Pharmacological premedication**	**Yes n. 40 (38%)**		**None n. 64 (62%)**
	Benzodiazepine n. 38 (95%)	Dexmedetomidine n. 2 (5%)	
**Airway devices**	**Endotracheal Tube** **n. 0 (0%)**	**Laringeal mask** **n. 23 (22%)**	**External device** **n. 81 (78%)**

^a^ Center with no preferent sedation or with use of multidrugs association (even both volatile and intravenous agents)

### NICU newborn sedation Techiques

Pharmacological sedation is preferred in NICU patients in 66% of cases but premedication rate was 18%. (Table 5) Neonatologists are responsible of 61% of non-pharmacological approach. Intravenous drugs and halogenated agents are used respectively in 30 and 49% of cases. However, when a neonatologist is involved in pharmacological sedation the use of sevoflurane is reduced to only 1 center. Conversely when the physician is only an anesthesiologist, sevoflurane is preferred to intravenous drugs (82% vs 10%). External device for the airways is preferred even most of time but in 9% of center the first choice is the endotracheal intubation.

**Table 5:** First choice Sedation technique in NICUcenters

Sedation in NICU centers n. 65			
**Drug Sedation**	**Yes n. 43 (66%)**		**None n. 22 (34%)**
	Sevorane n. 21 (49%)	Midazolam n. 9 (21%)	
	Thiopental n. 4 (9%)	Multidrugs^a^ n. 9 (21%)	
**Pharmacological premedication**	**Yes n. 8 (18%)**		**None n. 57 (82%)**
	Benzodiazepine n. 8 (100%)		
**Airway devices**	**Endotracheal Tube** **n. 4 (9%)**	**Laringeal Mask** **n. 3 (7%)**	**External device** **n. 36 (84%)**

^a^ Center with no preferent sedation or with use of multidrugs association (even both volatile and intravenous agents)

## Discussion

After the warning of the American Food and Drug Administration in the 2016 about the potential negative effects of general anesthetics and sedation drugs on developing brain [15], and the subsequent prompt reaction by many Societies of Anesthesiologists [16, 17], unicity of MRI setting has been the ideal “battlefield” to deal with this issue. Moreover, we are witnessing a change in literature: no more papers about techniques, but reviews about strategy to minimize sedation in pediatric MRI. Despite author’s efforts, the quality of evidence has not increased in the last years.

The need of a Survey focused on these topics comes from all the above mentioned considerations and follows the heels of two previous national Surveys, carried out in the United Kingdom [18] and in the United States [19]. The first one sadly lacks of details, and the second one considered a sample of only 58 tertiary NICU of the whole country. On the contrary, our data present more widespread, including low profile centers too. The investigation we promoted meant to be a step forward in the analysis of the main aspects involved in MRI management. Despite of the limitations of a phone survey (contact center response could not match exactly with objective data), the emerging picture raises the concern that there is still an extensive room for improvement*.*

The first data to comment is a fragmented nature of centers activity, with a large amount of low volume Institutions, where the number of procedures performed would not allow an adequate training and skilling of the teams involved. In the face of many Institutions working whit low-volume, there are only 26 centers (mostly University and/or Pediatric Hospitals) guaranteeing a flow of almost 10 pediatric patients a week. In the same way, among the 65 centers able to treat NICU’s neonates, only 12 do more than 3 weekly procedures on that range of age.

MRI for NICU’s patients involves neonatologists in a large proportion (40% vs anesthesiologists 58%), e and just in a few cases (2%) a cooperation with anesthesiologists is provided. The volume of neonates treated does not modify the rate of neonatologists (40% in HV centers and 41% in LV center). Unfortunately, we have no data about the presence of residents in the site.

Anesthesiologists look after children in almost all sites, independently of a NICU staff. Very few centers entrust this service to pediatricians, so the assistance of children and neonates in MRI largely rely on Anesthesia Services, which should always offer high levels of skillness and safety.

Looking at the data of the Survey, the centralization of pediatric activity tabled by National Anesthesiologists Societies (SIAARTI) and SARNePI in shared Documents [14], is an aim only partially achieved, and MRI for pediatric patients is still too fragmented in the Italian Hospitals.

A dedicated nurse is absent in almost a quarter of the Centers included in the Survey, but surprisingly the lack of a specific nurse assistance is greater when NICU’s patients are treated. Probably neonatologist support and/or NICU nurses accompanying the patients, would explain the difference.

IN, OUT and DH access to MRI are the usual ways to manage young patients for MRI. IN and DH are equally the commonest, and the choice of these models is probably due to the possibility of a postprocedural monitoring after sedation, even if data from literature do not suggest a safer profile with these models of care [20].

The respondents to this survey were also asked about monitoring and availability of specific devices. Despite several international recommendations [21], adverse events analysis [22], and national guidelines [23], the equipment is often obsolete and incomplete. The data emerging by the Survey confirm a general “technological” inadequacy in a setting otherwise so complex and challenging.

The results showed an extensive use of pulse oximetry and EKG [24], not only inside the MRI suite. Remarkably, EndTidal capnography is not used in quite 20% of the Centers, despite deep sedation is the technique of choice. While pulse oximetry is not reliable to detect promptly respiratory depressions occurring during deep sedations, capnography would be able to recognize a condition of hypoventilation and apnea. Recent guidelines mandate the implementation of Capnography for moderate-to-deep sedation both in adults and pediatric patients [25]. Respiratory complications are the commonest adverse events in pediatric/neonatal anesthesia and their prevention is recommended, especially in Non Operating Room Anesthesia settings [26].

Hemodynamics control is based mainly in EKG because the non-invasive blood pressure monitoring is unavailable (often for the lack of adequate sizes) in almost 40% of centers. This absence could result life threatening, primarily considering the wide use of drugs as propofol or dexmedetomidine [27], which have a significant impact on mean blood pressure. Literature strongly suggests that a little control on this value can worsen the outcome of children and neonates [28, 29].

A complete monitoring is available in just over half of centers (*n* = 68/106, 64%). This data is improved in correlation with: HV vs LV pediatric centers (*n* = 19/26, 73% vs *n* = 45/80, 56%); HV vs LV NICU centers (*n* = 10/12, 80% vs *n* = 33/53, 53%); the specialty of NICU performer (Anesthesiologist 74% vs Neonatologist 50%). It is difficult to explain the reason of such a limited monitoring, even in centers with HV MRI activity. Moreover, dealing neonates do not improve the availability of monitoring devices. It is out of doubt that expensive non-magnetic devices are often not available for economic restrictions, but a cultural issue is to be considered, which involves primarily the role of the Chiefs of the Anesthesia Services and Departments. Actually, they should be the first movers for an outstanding anesthesiological support.

If a compatible MRI ventilator is present in almost all cases, curiously the availability of vaporizers for Sevoflurane is not equally confirmed, as it is absent in a percentage varying between 14 and 18% of the Centers. Sevoflurane is the first-choice agent for the induction in uncooperative pediatric patients [30], and its deficiency restricts pharmacological choices to intravenous drugs, increasing the difficulty to manage the young patients, above all during the induction phase. Moreover, many MRI suites have not an adequate room scavenging system for halogenated (only 72% of centers are equipped with scavenger systems), causing a dangerous environmental pollution, above all in HV Centers. Furthermore, we have no data about potential use of halogenated agents in adjoining room to inhalation induction.

Also, the option to administer intravenous drugs are inadequate. Less than 40% of MRI rooms are indeed equipped with syringe pumps for intravenous infusion in a magnetic environment, obliging the use of single shot drugs or repeated boluses. To face this lack, it is quite common to use an external common pump outside the MRI room, connected to the patient by many extension sets [31]. A good alternative would be the use of traditional syringe pumps allocated into non-magnetic boxes, which allow the anesthetist to infuse sedative drugs near the patient [31].

In the 11% of centers there are neither halogenated vaporizers nor infusion pumps. The general sensation is disappointing because the traditional drawbacks of NORA apply totally to a qualified and critical setting such as MRI in pediatric age. Obsolete and incomplete devices and monitoring systems, increase the risks of adverse events in far environments where anesthetists work alone, without expert personnel supporting them [32].

Pharmacological approach is undoubtedly preferred for children and for NICU’s patients too when the physician in charge is an anesthesiologist, with a leading role played by sevoflurane. Intravenous drugs are common mostly when vaporizer is not available and when the performer is a neonatologist, but the survey does not show a clear indication about a preferred combination. The lack of dexmedetomidine use caught our attention, mostly in neonatal age. Its role as only one medication which should not has negative effect in neurological development [33] did not affect clinical practice.

Benzodiazepines are the most common agent for premedication (and the only one used in neonates). A low-invasive airways management is widespread: laryngeal masks are under-utilized, and tracheal intubation is not a first choice in children and an exceptional standard in neonates.

This study has several limitations and at the same time much food for thought. Our phone survey investigated a single western Country experience and contacted the physician responsible of sedation, anyway we deemed it could be a starting point to monitor the further evolution of approach to children and neonates in MRI suite. We described a diversified model of organization, with an extreme variation in in/outpatients pathways. Unfortunately, our information is not sufficient to identify the best option, and this aspect was not an objective of our survey. Although it would be interesting to verify the degree of expertise of providers, we considered this survey an inadequate tool to investigate this item.

The current rate of a dedicated nurse supporting the performer is still unsatisfactory, but we have no data about a possible involvement of residents.

For which concern monitoring and tool Italian centers need to quick improve their supplies, which in many cases are clearly below the threshold of Minimum Standard [34]. Further analysis of our data will be focused on this topic to evaluate if there is a correlation between monitoring and tools availability and sedation choices.

## Conclusions

NORA represents an increasing activity, but quality and safety are two essential goals of our practice. Our survey shows a fragmentated frame of organization probably reflecting the needs of adapting to different local requirements. Availability of tools and devices, the skilling of performer, and the presence of helping staff influence sedation choices.

The aim of our survey was to offer a realistic picture of the “state of the art” in order to promote a more qualified approach. Italian frameworks rely on the daily experience and practice, as our data show, but we need a clear indication by statements and recommendations by National and International Societies. Unfortunately, many aspects are not sufficient supported by actual evidence to lead the way. We hope that our results could be useful for stimulate further research in this field.

## Supplementary Information

**Additional file 1.** Survey Complete: Logistic organization, Pediatric management and NICU’s neonates management.

## Data Availability

The data that support the findings of this study are available from the corresponding author upon reasonable request.
