# Supplementary material for: Children and neonates anesthesia in magnetic resonance environment in Italy: an active call survey
Source: BMC Anesthesiol. 2022 Sep 2;22:279. doi: 10.1186/s12871-022-01821-3 (PMC9438255; doi:10.1186/s12871-022-01821-3)
Supplement: Supplementary file 1 — Additional file 1. Survey Complete: Logistic organization, Pediatric management and NICU’s neonates management. [file 12871_2022_1821_MOESM1_ESM.docx]

**Children and Neonates Anesthesia in Magnetic Resonance Environment in Italy.**

**An active Call Survey**

*Center………………………………………………………………………………………………..*

*Reference Person ……………………………………………………………………………………………*

*Contact data (mail/phone)………………………………………………………………………………….*

*Investigator………………………………………………………………………………………*

*Rome __ / __ / __ Signature …………………………*

**Section I Logistics**

1. **Procedures**

1.1 How many paediatric procedures do you think are performed a week?

1.2 Rate in % of inpatient ___%; day hospital __%; outpatient __%

1.3 How many NICU procedures do you think are performed a week?

1.4 Who is usually the performer for children (0-14 years old)?

- Is there a dedicated nurse help?

1.5 Who is usually the performer for NICU neonates?

- Is there a dedicated nurse help?

1. **Monitoring available**

Inside Outside

2.1 Saturimetry ______ _______

2.2 EKG ______ _______

2.3 EtCO_2_ ______ _______

2.4 NiBP ______ _______

**3. Tools available**

3.1 Aspiration Tube yes no

3.2 Mechanical ventilator yes no

3.3 Halogenated vaporizers yes no

3.4 Scavenger Systems yes no

3.5 Amagnetic infusion pumps yes no

**Section II – First Choice Technique in Paediatric Patients (0-14 years old)**

Non Pharmacological approach

Pharmacological approach with external device for airways management

- Premedication

NO

YES (which drugs?)

- Sedation Drug Maintenance

Halogenated

Propofol

Benzodiazepine

Opioids

Tiopenthal

……

- Airways Device

Nasal Probs

External Mask

None

Pharmacological approach with internal device for airways management

- Premedication

NO

YES (which drugs?)

- Sedation Drug Maintenance

Halogenated

Propofol

Benzodiazepine

Opioids

Tiopenthal

……

- Airways Device

Endotracheal tube

Laryngeal mask

….

**Section III – First Choice Technique in NICU Patients**

Non Pharmacological approach

Pharmacological approach with external device for airways management

- Premedication

NO

YES (which drugs?)

- Sedation Drug Maintenance

Halogenated

Propofol

Benzodiazepine

Opioids

Tiopenthal

……

- Airways Device

Nasal Probs

External Mask

None

Pharmacological approach with internal device for airways management

- Premedication

NO

YES (which drugs?)

- Sedation Drug Maintenance

Halogenated

Propofol

Benzodiazepine

Opioids

Tiopenthal

……

- Airways Device

Endotracheal tube

Laryngeal mask

….
